# Assessment of Brushing Techniques in School Children and Its Association with Dental Caries, Omdurman, 2019

**DOI:** 10.1155/2021/4383418

**Published:** 2021-01-22

**Authors:** Rugaia Eltayeb Hag Maki Ibrahim, Maha O. Helaly, Ehab Mohamed Abdelhlim Ahmed

**Affiliations:** ^1^Department of Pediatrics, Faculty of Dentistry, University of Medical Science and Technology, 79371, Khartoum, Sudan; ^2^Department of Restorative Dentistry, Faculty of Dentistry, University of Medical Science and Technology, 79371, Khartoum, Sudan

## Abstract

**Background:**

Dental caries are a common infectious disease of childhood. It is a highly prevalent disease among children.

**Aim:**

The goal of this study was to assess the different brushing techniques used by school children and to identify if there is an association between brushing techniques and dental caries.

**Materials and Methods:**

A descriptive cross-sectional study was carried out among 396 school children (9–12 years old) chosen by convenience sampling technique from schools located in Omdurman locality, Sudan. After consent was taken, students were asked in an interview-based questionnaire about their brushing habits. Consequently, they were examined in an upright position using a sterile mouth mirror and a probe. The examination was carried out by a single examiner to investigate the presence of dental caries. The diagnosis was done based on the World Health Organization diagnostic criteria 2013. The data were analyzed through descriptive statistics and comparison between values using the chi-square test with a *P* value <0.05.

**Results:**

The present study found that the prevalence of dental caries is 70.9%. The combined brushing technique was the most used technique (42.9%). There was no statistical significance between brushing techniques and dental caries (*P* value ≤0.330). There was a statistical significance between the periodic change of the toothbrush and dental caries (*P* value ≤0.001). There was statistical significance between the level of education of the mother (*P* value ≤0.001) and father (*P* value ≤0.012) to the brushing technique used by the child as well.

**Conclusion:**

Due to a lack of awareness consequent of lower socioeconomic status, only a few percentage of the Sudanese population knows how to implement the correct oral hygiene practices to combat dental caries. It is important to design a specific public health program that particularly targets families of low socioeconomic status, which represents the majority. Dental caries persist as a widespread condition in Sudan as a result of a deficit in these kinds of programs.

## 1. Introduction

Dental caries are an oral health condition that results from plaque accumulation on the tooth surface, converting sugar found in food and drinks into acid, consequently resulting in tooth damage [1]. It is considered a major health problem affecting 2.4 billion people worldwide in the year 2010 [2]. It is linked to poor oral hygiene, diet, and infrequent dental visits [3]. Dental caries are a widespread health problem among children [4], prevailing around 60–90% [5]. Moreover, dental caries influence a child's quality of life psychologically [5] exposing him/her pain and discomfort at an early age. Children with caries were found to be 1.3 times more likely to experience oral health-related impact on the quality of their lives [6].

With all the aforementioned implications of dental caries, the necessity of tackling it becomes evident. To understand the high prevalence, one must consider the different risk factors [5]. By identifying them, caries can be controlled and new preventive measures can be attained [5].

One of the risk factors of caries is inadequate oral hygiene practices [4]. Several studies show an association between dental caries and brushing habits [7]. An explanation for this association is the constant removal of plaque accumulation as a result of frequent brushing reduces the chances of dental caries [1].

About 31% of children in Nepal that suffered oral pain and high decay index had poor brushing habits [7]. In Saudi Arabia, brushing frequency was found to be associated with dental caries [8].

Similarly, in Abu Dhabi, United Arab Emirates, infrequent brushing was also found to be associated with dental caries [9]. Sudan is one of the countries that suffer from a high prevalence of dental caries among children [10, 11]. Therefore, we assume one of the possible causes of the high prevalence of caries in Sudan is brushing habits.

This research aims to evaluate and assess various tooth-brushing techniques and their association with dental caries among school children (9–12 years old) in Omdurman locality, Sudan. This research not only substantially expands the scope of research concerning this topic in Sudan but also provides information about dental caries in Omdurman, a locality whose inhabitants originate from many diverse backgrounds and tribes, consequently, reflecting information about dental caries in a larger sector of Sudanese society. Conducting a research investigating the cause of the high prevalence of dental caries in Sudan will help in tailoring prevention measures most suitable for the country.

## 2. Materials and Methods

A cross-sectional study was conducted to investigate the most used brushing techniques and their association with dental caries. This study was carried out on schoolchildren aged 9–12 years old, of both sexes, living in Omdurman, Sudan. Due to the cognitive development of children during the ages 6–12, children can think in concrete ways about objects and events. Therefore, they would give more reliable, accurate information about their brushing habits compared to their younger counterparts [12]. Another factor that contributed to choosing this age group is the nature of education in Sudan. Compulsory education lasts 8 years for ages 6–13 [13]. Therefore, 12-year olds make up a larger sector of students in schools compared to other age groups [14, 15]. The inclusion criteria were the age group, health, and residency of participants in Omdurman locality. The exclusion criterion was dental anxiety. The sample size was calculated from the total number of schools in Omdurman locality, where there were 280 private schools and 216 public schools. The total number of students aged 9–12 years was found to be 33,695. The sample size was found to be 396 students and was computed using the known population sampling equation:

(1) $$\begin{array}{c} n=\frac{N}{1+N{\left(e\right)}^{2}}, \end{array}$$

where *n* was sample size to be computed, *N* was the known population size, and *e* was the degree of accuracy desired (or accepted margin error and is usually set to 0.05).

The 198 students were selected from each school (governmental and private) using convenience sampling. Due to the unstable political climate of the country at the time the research was conducted, schools were constantly closing to ensure the safety of the children and hence data collection using other sampling techniques was difficult to implement.

Ethical clearance was obtained from the Ethical Committee at the Faculty of Dentistry at the University of Medical Science and Technology. Local authorities (the Ministry of Higher Education and Research and Ministry of Education in Khartoum State) provided the names, contact information, location, and the number of schools and students in both private and governmental schools in Omdurman locality. Written consent letters were given to all parents clearly expressing the aims and objectives of this study and asking for voluntary participation of their child for examination with no complication on withdrawal. The letter included information about the nature of the questionnaire as well. The consents were signed by parents who agreed to participate. Informed consent from each student was obtained before the oral examination.

Data collection was done through an interview-based questionnaire and followed by an oral examination. The interview-based questionnaire involved questions regarding personal information, brushing techniques, brushing frequency, how often are their toothbrushes changed, if any other brushing aids are used, and toothbrush type. The oral examination was done by a single examiner, who is the main author. It was carried out in the teachers' office where participants were exposed to natural light. Each student was seated in an upright position. Using disposable masks and gloves, participants were examined with a sterile plain mirror and a sterile dental probe. Before collecting the data, the author was calibrated for the clinical parameter. The kappa value was found to be 0.9 and 0.8 for a questionnaire and clinical parameter, respectively. The kappa values were considered almost perfect according to the qualitative classification of kappa value.

Any tooth with a lesion on a smooth tooth surface or a pit or fissure with a detectably softened floor, undermined enamel or softened wall, tooth surface containing temporary filling requiring further treatment, a tooth surface containing a permanent restoration with an area of decay (either primary or secondary caries) was considered a carious tooth. Caries severity was measured for permanent teeth by DMFT (decay, missing, and filled) index, which records the number of *D* (decayed tooth). The DMFT score was recorded according to the WHO oral health assessment form for children in 2013 as follows [16]:

0 = sound, 1 = caries, 2 = filled with caries, 3 = filled, no caries, 4 = missing due to caries, and 5 = missing for any other reason.

After the examination was performed, participants were given oral health instructions regarding brushing habits and dental caries. Data were entered and analyzed using Statistical Package for Social Science (SPSS) version 20. The qualitative variables were displayed using frequencies and percentages. Quantitative variables were displayed using mean and standard deviation. The chi-square test was done to determine the association between brushing technique and dental caries, and statistical significance set at *P* < 0.05. Bivariate regression analysis was performed to check for the relation between brushing habits and dental caries.

## 3. Results

A total of 396 school children aged 9–12 years living in Omdurman locality, Sudan, participated in this study. The distribution of the sample size is shown in Table 1.

Through the data collected from the sample, information regarding brushing habits and frequency of practice amongst participants was determined. About 96.5% of the participants cleaned their teeth; moreover, 93.4% of those who cleaned their teeth used toothbrush and toothpaste to maintain oral hygiene. Participants who changed their toothbrushes were found to be 93.4%. About 47.2% discarded their toothbrush when useless, 36.7% once every three months, and six-point-six percent did not change their toothbrush. The information is found in depth in Tables 2 and 3.

The combined brushing technique was the most used technique (42.9%). The horizontal brushing technique (32.6%) was the second most used technique followed by the circular brushing technique (15.2%). The vertical technique was the least common (9.3%). Moreover, the most used brushing technique deployed by males and females was horizontal and combined techniques, respectively. The overall prevalence of caries was found to be 70.9%.

There was no statistical significance between the technique used for tooth-brushing and dental caries (*P* value ≤0.330). However, there was a statistical significance between the level of the education of the mother and the brushing technique deployed by the participants (*P* value ≤0.001). Similarly, statistical significance was found between the level of education of the father and the brushing technique (*P* value ≤0.0012).

There was a statistical significance between dental caries present in participants and their mothers' and fathers' level of education (*P* value ≤0.001; *P* value ≤0.012), respectively.

Regression analysis revealed that participants who changed their toothbrush when useless (*β* = −1.027, *P*=0.012, OR = 0.358, and CI = 0.160, 0.801) and every three months (*β* = −1.511, *P*=0.000, OR = 0.221, and CI = 0.098, 0.497) were less likely to develop dental caries than those who changed their toothbrush every six months. In addition, participants whose mothers only completed secondary school (*β* = 0.805, *P*=0.017, OR = 2.237, and CI = 1.152, 4.345) were of higher risk of dental caries than those whose mothers completed university. While participants whose fathers completed less than primary or only primary school (*β* = 1.323, *P*=0.017, OR = 3.756, and CI = 1.270, 11.113) were of higher risk of caries than those whose fathers completed university. Finally, males (*β* = 0.663, *P*=0.003, OR = 1.941, and CI = 1.247, 3.021) were almost twice as likely to have dental caries than females (Table 4).

## 4. Discussion

The prevalence of dental caries is not only influenced by biological factors that interact with the causative microorganisms, but it is also associated with socioeconomic status, educational conditions, and dietary habits [13]. As a result of the multiple factors that contribute to its causation, dental caries is one of the most common diseases not just locally but globally. This increased prevalence increases the concerns and motives to explore and discover new measures that will aid in its control. In this study, an interview-based questionnaire was done and each student was examined using the WHO diagnostic criteria 2013. A total of 396 participants was included with a response rate of 100% (*n* = 396). The results have shown that the prevalence of caries found among the age group 9–12 years was about 70.9%. The combined technique was the most used brushing technique.

When the prevalence of caries in this study compares to the prevalence reported throughout the past years in Sudan, a rise in dental caries can be noticed. A study conducted by Nurelhuda on the oral health status of 12-year-old schoolchildren in Khartoum State, Sudan, a school-based survey, stated that “in 1966, the DMFT of 10- to 14-year-old children was reported to be 0.7 in Sudanese children generally and 1.5 in Khartoum Province/State specifically. In 1986, Ibrahim et al. reported DMFT values of 6- to 13-year olds, in three areas within Khartoum Province, classified by the authors to urban, semiurban, and rural, of 2.9, 3.2, and 2.3, respectively [3]. Sampling procedures were not clarified. Two years later, this was followed by a reported rise of DMFT to 3.2, among a randomly selected sample of school children in the Omdurman locality [4]. These authors expressed concern towards an “alarming rise” which suggested that we might find an even higher DMFT value. The present study results indicate a decline” [13]. The results of the current study prove the alarming rise predicted in the earlier study. This rise can be due to deteriorating dental health care, lack of awareness, or an increase in poverty rates on a national level.

When the prevalence of caries in Sudan is compared globally, it was found that Sudan has a higher caries prevalence (70.9%) compared to that reported in Al-Hassa, Saudi Arabia (68.9%) [16]. On the other hand, it was found lower yet similar to the prevalence of caries in Egypt (74%) [17], India (77%) [15], and Riyadh, Saudi Arabia (83%) [18]. From these findings, we can observe an underlying pattern that suggests a possible link between the prevalence of caries and a country's Global Domestic Product (GDP) taking into consideration other factors such as the difference in time in which the research was conducted, the date of the publication of the research, and other variables that may have influenced the research. A study on global epidemiology of dental caries and severe periodontitis, a comprehensive review, reported that the prevalence and severity of cavitated dentine carious lesions among 5–12-year olds have declined over the last decades and a lower prevalence was reported among 12-year olds and 35–44-year olds specifically in high-income countries.

Moreover, the number of teeth present in elderlies has increased over the last four decades and this might be a result of fewer teeth being extracted because of dental caries. Findings from the WHO database on dental caries epidemiology include a table that displays the association found between dental caries and a country's income [19].

Countries with low income have a high prevalence of dental caries. On the contrary, countries with a higher income have a lower prevalence of caries. One of the possible reasons behind this association can be the lack of oral health personnel. According to the WHO, the dentist to population ratio in Africa is around 1 : 150000 while most industrialized countries are estimated to be around 1 : 2000 [20]. Another explanation is the difference in the economic growth of countries, availability of resources, general socioeconomic status, dietary habits, and lastly knowledge about brushing techniques and dental caries.

This study investigated the most common brushing habits among children to gain information about participants' knowledge of dental care and its association with dental caries. Results have shown that the most used technique was the combined brushing technique (42.9%), followed by the horizontal (32.6%), circular (15.2%), and lastly the vertical technique (9.3%). There was a negative association between the brushing technique used and dental caries. These findings contradict the previous presumptions on the possible correlation brushing techniques and dental caries. In accordance with the findings of a study conducted in Abu Dhabi, this study has found a negative association between brushing frequency and dental caries [9]. However, one study conducted in Saudi Arabia reported a correlation between brushing frequency and dental caries [8, 21]. On the other hand, there was a strong association between the frequencies of toothbrush change to dental caries. The longer the toothbrush use, the weaker and less efficient the toothbrush bristle effectively remove bacteria [22]. In this regard, the country's economic conditions impact negatively. Moreover, the socioeconomic status of families affects the ability to change the toothbrush periodically.

Another finding of this research is the frequency of brushing practiced by the participants. About 15.9% brushed two or more times a day while almost 55.8% practiced brushing only once daily. It is concerning that the participants who brushed two or more times a day only constitute a minority. This is further evidence of the lack of information on good oral health practices in Sudanese communities. A survey taken in 2012 on oral health habits among Sudanese population showed a depressed point of awareness in the nation [23]. Despite the eight-year gap between the aforementioned research and current research, the conclusions drawn about awareness are alarming.

The level of education of the parents is a strong indicator of many factors such as their socioeconomic status and knowledge on the importance of oral health maintenance therefore affecting the children of the community. There was a positive association between the periodic change of toothbrush and the parents' level of education. There was a positive association between dental caries and the level of education of the parents as well. A survey conducted in Sudan on the knowledge and practice of mothers in relation to dental health among preschool children has found an association between dental health knowledge and practice to the mother's educational level [22].

A study on dental caries and existing oral health care of children and adolescents in Hubei Province, People's Republic of China, reported similar findings of a high prevalence of untreated dental caries in studies conducted on school children who had limited access to dental care [21]. One of the possible reasons behind the limited access to dental care can be low socioeconomic conditions. Some of the families of higher educational levels have higher socioeconomic conditions and can afford regular dental visits from which they can learn more about the oral health importance and oral health instructions thereby possessing a higher awareness and knowledge of oral health and care.

Dental caries is not just a prevalent disease, but it also exerts high pressure on the country's economy. According to the World Health Organization, dental caries is the fourth most expensive chronic disease group to treat [24]. Dental caries, therefore, places a major financial burden on both individuals and health care systems [25].

This study provides a clear relationship between socioeconomic factors, dental caries, and the country's economy. This cycle will persist as long as these factors are not put into consideration when devising a public health program against dental caries. Therefore, this research creates a valuable addition to ones' knowledge of dental caries and awareness level in Omdurman, Sudan, and provides information necessary to its resolution. On the other hand, the limitation of this study is the convenient sampling technique used. We advise future researchers to use other sampling techniques.

## 5. Conclusions

In conclusion, all of the previous findings can be explained by the low levels of awareness and education on oral health. Knowledge and awareness are very important tools that are underestimated in Sudan and more specifically Omdurman when fighting a highly prevalent disease such as dental caries. One of the reasons behind the exponential rise of dental caries in the past years has been negligent of the importance of awareness and its relationship to dental caries, the country's economy, and the families' socioeconomic status. Oral health programs should be organized to spread awareness on dental caries, the importance of brushing, and dental visits. Furthermore, these programs should place an emphasis on families of low socioeconomic status.

## Figures and Tables

**Table 1:** Distribution of the sample size according to age, gender, grade, and education.

Variables	*N*	%
Age		
9	29	7.3
10	113	28.5
11	194	49.0
12	60	15.2
Gender		
Female	195	49.2
Male	201	50.8
Grade		
5^th^ grade	196	49.5
6^th^ grade	200	50.5
Education		
Governmental	198	50.0
Private	198	50.0

**Table 2:** Distribution of the sample size according to the number of participants who practiced brushing.

Variables	*N*	%
Participants who practiced brushing		
Yes	382	96.5
No	14	3.5

**Table 3:** Distribution of the sample size according to brushing habits: brushing tools, brushing frequency, toothbrush type, and brushing technique.

Variables	*N*	%
Brushing tools		
Tooth brush and toothpaste	37	93.4
Toothbrush, toothpaste, and floss	12	6.6
Brushing frequency		
Several times a week	112	28.3
Once a day	221	55.8
Two or more times a day	63	15.9
Toothbrush type		
Soft	98	24.7
Medium	231	58.3
Hard	67	16.9
Brushing technique		
Circular	60	15.2
Horizontal	129	32.6
Vertical	37	9.3
Combined	170	42.9

**Table 4:** Regression model of the association between brushing habits (brushing technique, brushing frequency, and toothbrush changing frequency), parents' level of education, gender, education, and DMFT (decayed, missing, and filled).

Variables	Regression coefficient (*β*)	*P* value	Odd ratio	95% CI

Brushing technique				
Circular				
Horizontal	0.097	0.789	1.102	(0.539, 2.253)
Vertical	−0.255	0.587	0.775	(0.309, 1.944)
Combined	−0.453	0.182	0.636	(0.327, 1.237)
Constant	1.076	<0.001	2.933	
Brushing frequency				
Several times a week			
Once a day	−0.498	0.062	0.608	(0.360, 1.026)
Two or more times a day	0.754	0.077	2.125	
Constant	0.981	<0.001	2.667	(0.922, 4.899)
Toothbrush change frequency				
Once every six months				
When useless	−1.027	0.012	0.358	(0.160, 0.801)
Once every three months	−1.511	<0.001	0.221	(0.098, 0.497)
Constant	1.981	<0.001	7.250	
Mother's level of education				
University completed				
High school completed	−0.142	0.635	0.867	(0.481, 1.562)
Secondary school completed	0.805	0.017	2.237	(1.152, 4.345)
Primary school completed	0.263	0.397	1.301	(0.708, 2.391)
No formal school or less than primary school completed	0.117	0.869	1.124	(0.280, 4.521)
Constant	0.730	<0.001	2.075	
Father's level of education				
University completed				
High school completed	−0.123	0.641	0.885	(0.528, 1.482)
Secondary school completed	0.243	0.428	1.275	(0.699, 2.324)
Less than primary school or primary school completed	1.323	0.017	3.756	(1.270, 11.113)
Constant	0.787	<0.001	2.196	
Gender				
Female				
Male	0.663	0.003	1.941	(1.247, 3.021)
Constant	0.580	<0.001	1.786	
Education				
Governmental				
Private	0.221	0.319	1.247	(0.807, 1.927)
Constant	0.565	0.103	1.759	

## Data Availability

The data are available from the Ministry of Education in Omdurman locality, Sudan.
